# The Discovery of Small-Molecule Mimicking Peptides through Phage Display

**DOI:** 10.3390/molecules16010774

**Published:** 2011-01-19

**Authors:** Fahriye Ceyda Dudak, Ismail Hakki Boyaci, Brendan P. Orner

**Affiliations:** 1 Department of Food Engineering, Hacettepe University, Beytepe 06800, Ankara, Turkey; E-Mails: ceyda@hacettepe.edu.tr (F.C.D.); ihb@hacettepe.edu.tr (I.H.B.); 2 Division of Chemistry and Biological Chemistry, Nanyang Technological University, 21 Nanyang Link, Singapore 637371, Singapore

**Keywords:** phage display peptides, biotin mimics, sugar mimics

## Abstract

Using peptides to achieve the functional and structural mimicry of small-molecules, especially those with biological activity or clear biotechnological applications, has great potential in overcoming difficulties associated with synthesis, or unfavorable physical properties. Combinatorial techniques like phage display can aid in the discovery of these peptides even if their mechanism of mimicry is not rationally obvious.The major focus of this field has been limited to developing biotin and sugar mimetics. However, the full “mimicry” of these peptides has not yet been fully established as some bind to the target with a different mechanism than that of the natural ligand and some do not share all of the natural ligand’s binding partners. In this article, mimicry of small-molecules by phage display-discovered peptides is reviewed and their potential in biochemical and medical applications is analyzed.

## 1. Introduction

Phage display has been employed to identify ligands that specifically bind to almost any target. Therefore it is possible to design phage display screens to discover peptides that dock selectively to protein binding sites and that are competitive with the natural ligands. If these peptides can interact with many of the protein receptors that bind the natural ligand then the peptide can be considered mimetic. If the natural ligand is a small-molecule then the phage-selected peptides are mimics of the small-molecule. An especially interesting molecular recognition characteristic of this is that the small-molecules and their mimetic peptides are often very different chemical classes of compounds, and the mimicry is rarely obvious upon comparing their chemical structures. This latter fact makes the rational design of a peptidic small-molecule mimic nearly impossible thereby necessitating the use of a combinatorial technique like phage display. 

Natural small-molecule ligands can have multiple disadvantages in that they can be difficult to chemically synthesize, they may be challenging to work with due to unfavorable physical properties, or, due to synthetic limitations, it may be unwieldy to completely and rapidly explore structure-activity relationships or to rationally or combinatorially advance them to molecule classes with more favorable properties. Peptides, on the other hand, are usually trivial to synthesize or produce molecular biologically. They can be generated as part of protein fusions and are amenable to applications requiring the employ of bioconjugation strategies. In addition, they tend to be immunogenic and can therefore be used for the generation of antibodies against the natural ligand. Moreover, if the small-molecule acts as an enzyme substrate, the mimetic peptides may not be reactive when bound to the enzyme active site and therefore they would act as inhibitors due to their lack of “chemical mimicry”. Finally, there are many strategies for their advancement to peptidomimetics, thereby formally achieving the transformation of a ligand from one small-molecule class to another.

This review will focus on the phage display discovery of peptides mimicking specific non-peptidic small-molecules. This field has been restricted to mainly mimics of biotin and sugars. The structural similarities between these molecules and the peptide mimics will be discussed as well as their functional properties. A better understanding of the relationship between structural and functional mimicry, and the design of more specific screens, may facilitate the design and optimization of future peptide mimics derived from phage display, and the application of this philosophy in general, to other classes of small-molecules.

## 2. Biotin Mimics

One of the most successful and widely used class of peptide-based, functional mimics of small-molecules discovered through phage display screening are the biotin mimics. The noncovalent interaction between biotin, a small-molecule vitamin, and streptavidin, a tetrameric protein isolated from *Streptomyces avidinii*, is among the strongest known and has been the basis of countless biotechnologies. Avidin, another biotin-binding protein isolated from egg white, is structurally related and has a similar affinity to streptavidin, despite sharingonly 30% sequence identity [1]. However, the utility of avidin is somewhat limited due to its glycosylation and high pI resulting in binding non-specificity. NeutrAvidin, a deglycosylated form of avidinpossesses enhanced biotin binding specificity [2]. Peptide-based ligands that functionally mimic biotin could expand applications of streptavidin, avidin, and NeutrAvidin by providing sequences that when appended as fusions to expressed or synthesized proteins and peptides could be used for affinity purification or immobilization. In addition, as the affinity of biotin is often too strong for competitive release, it might be possible to tune the affinity using peptide ligands either rationally or through screening. The utility of peptide-based biotin functional mimics is therefore clear, however what is not as evident is how to design this function into a polypeptide. Thus, combinatorial methods like phage display have provided flexible means to discover such peptide ligands.

The first phage display-based strategy to discover peptides which mimic biotin, was developed by Devlin *et al.* [3] who used a 15-mer peptide library of 10^7^ clones expressed as fusions on the M13 pIII protein to screen against streptavidin. Among the twenty selected phage isolates resulting in nine unique sequences, all exhibited a His-Pro convergence. Seven of these contained a His-Pro-Gln (HPQ) sequence. Similar work was carried out with a pentapeptide library generated from split and pool synthesis [4]. These results confirmed those from the phage screen and resulted in twenty-three of the twenty-eight isolated peptides possessing the HPQ consensus. Moreover, competitive binding studies demonstrated that the sequence HPQ and biotin were recognized by the same streptavidin binding site supporting the notion that HPQ is indeed a functional mimic of biotin. The equilibrium affinity of the motif to streptavidin was first rigorously quantified by Weber *et al.* [5]. Employing titration calorimetry, this study explored the thermodynamics and structural mechanism of binding using 7-mer peptides having the common sequence HPQN. Analysis of the data suggested the formation of a 1:1 binding complex, however the dissociation constants of the peptides were found to be in the milimolar range whereas the natural ligand binds with femtomolar affinity. Although the binding is weaker, a crystal structure of a peptide-streptavidin complex showed that the side chains of the HPQ sequence were binding in the biotin binding pockets which are located at the ends of streptavidin β-barrels. 

In order to optimize binding and to further explore the design rules of biotin mimicry, Kay *et al.* [6] used a phage library displaying peptides with two eighteen random amino acid regions flanking a Pro-Gly dipeptide to screen against streptavidin. Among other reasons, it was thought that longer peptides might be able to stabilize a more favorable binding conformation, and presumably the Pro-Gly was included to facilitate this. Two classes of binding peptides were discovered. One shared the HP(Q/M) motif and the other had no clear consensus. Surprisingly, phage clones from the second class bound streptavidin with a higher affinity than the ones having the HPQ motif although both classes of peptides were competitive for the biotin-binding site. Moreover, synthetic 7-mer peptides having the HPQ motif were not able to inhibit the binding of the selected phage clones of either of the two classes, indicating that stabilized secondary structure may have been playing a role in enhanced binding. However, it should be born in mind that the affinities were determined and compared using whole phage with multiple copies of the peptide displayed on the capsid. The multivalent effect therefore may be playing a role [7,8]. The concept of optimizing the binding through enhanced structure was also used by McLafferty *et al.* [9] who demonstrated that the affinity of peptide ligands can be increased by constraining the peptides with disulfide bonds.A cyclic peptide 8-mer library with six random residues in the format of XCX_4_CX was panned against streptavidin and most of the selected peptides converged on the HPQ motif. Emphasizing the importance of the disulfide bond for imparting enhanced binding due to conformational preorganization, treatment with DTT significantly decreases the affinity of the phage clones relative to a known control binder containing no disulfide bond. It is expected, however that DTT may reduce some of the key structural disulfides present naturally in the phage capsid proteins. However, structural and thermodynamic analysis using similar synthetic ligands has confirmed that a decrease in both ligand and solvent entropy resulting in an increase in affinity can be realized by constraining the conformations of the linear peptides through cyclization [10]. Furthermore, Giebel *et al.* [11] screened six, seven and eight amino acid (CX_4_C, CX_5_C, CX_6_C) cyclic-peptide libraries against streptavidin and all selected sequences contained the HPQ motif. The affinities (K_D_ = 230 nM) of the synthetic cyclic peptides towards streptavidin were determined using a surface plasmon resonance (SPR) biosensor and were found to be three orders of magnitude higher than the corresponding linear peptides. Crystal structures of streptavidin-bound cyclic peptides [12] having the HPQ motif, demonstrate that the linear and cyclic peptides make common interactions with streptavidin suggesting that the effect of cyclization on affinity is primarily entropic. Following this logic, Katz *et al.* [13] attempted an additional optimization by replacing the disulfide cross-link with a thioether based on the reasoning that this linkage would avoid oligomerization and more easily permit the binding conformation. However, the resulting cyclic peptides exhibited affinities (K_D_= 680 nM) similar to those of the disulfide-constrained peptides. A further disadvantage, of course, is that it is unclear if these structures could be used in a phage library screen. With that said, displayed libraries have been created that conformationaly constrain peptides through cysteine residues which react with tribromo extrinsic crosslinkers by means of an SN2 reaction [14]. Therefore, it is conceivable that other mechanisms of constraint employing unique chemistries could be employed in future phage display libraries. Another optimization strategy in a similar philosophical vein was the use of the Trp cage mini-protein domain [15]. The Trp cage fold is stabilized by a hydrophobic pocket formed between a tryptophan, tyrosine, and a number of proline residues, resulting in a fold containing short α- and 3_10_-helicies.It should therefore have a relatively rigid structure prior to binding. A Trp cage-based library (DXYXQWLX_3_GPXSGRP_3_X) displayed on the lytic bacteriophage, T7, was panned against streptavidin and resulted in a number of HPQ-containing active peptides.

A true mimic of biotin should be able to bind all the receptors that bind biotin, however only a few studies have subjected their resulting ligands to this challenge, and most have failed. Roberts *et al.* [16] panned a 6-mer, linear phage display library against an anti-biotin antibody. The resulting matured library was able to converge on the sequence XXYYLH which is somewhat surprising considering that the antibody was polyclonal. However these peptides failed to bind streptavidin suggesting that they are not true biotin mimics. In another study, phage libraries of linear 8-mer peptides fused to the major coat protein, pVIII, (aka “landscape libraries”) were used to screen for high affinity ligands against streptavidin and NeutrAvidin [17]. It has been argued that landscape libraries, due to the fact that steric constraints influenced by display on every copy of the major coat protein, result in more rigidconformations of the peptides. Despite the similarity between the resulting sequence motifs discovered for streptavidin (VP(E/D)(G/S)AFXX) and NeutrAvidin (VPE(F/Y)XXXX), the phage selected for each target had no affinity for the other. Meyer *et al.* [18] used a phage displayed cyclic peptide library (CX_6_C) with the fusion displayed on a single copy of pIII to pan against NeutrAvidin. The selected peptides, containing the novel consensus motif CD(R/L)A(S/T)P(Y/W)C, bound both NeutrAvidin and avidin with disassociation constants in the low µM range. Although these peptides show similar affinities for both avidin and NeutrAvidin, they did not bind to streptavidin, again suggesting that they are not universal biotin mimetics.

**Figure 1:**
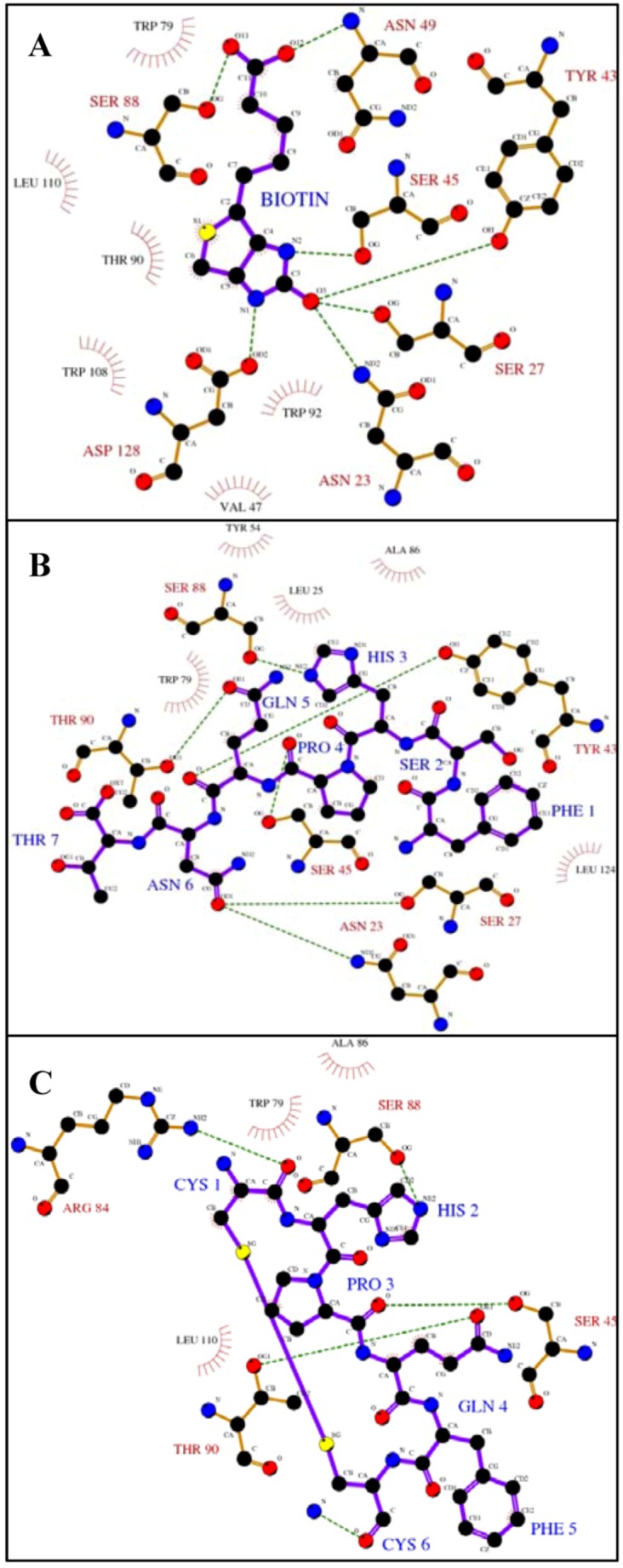
Ligplot analysis [25] showing the hydrogen bonding and hydrophobic interactions between streptavadin and biotin or peptides with the HPQ motif.The figures depict binding between streptavidin and **A)** biotin [26], **B)** a linear peptide, FSHPQNT [12] and **C)** a cyclic peptide, Ac-CHPQFC-NH_2_ [12]. Hydrogen bonds are shown as green dashed lines and hydrophobic interactions are shown as red arcs. Key protein residues are labeled in black (hydrophobic) or red (hydrogen bonds) and ligandresidues in blue. Because this is a two-dimensional representation, actual distances, bond lengths, and spatial organization are distorted.

The biotin binding site of streptavidin is lined with polar and aromatic amino residues (Figure 1A). Upon binding, multiple interactions, dominated by hydrogen bonds and Van der Waals contacts, occur between the biotin and the protein. The urea moiety of biotin makes hydrogen bonds with Asn 23, Ser 27, Ser 45, Tyr 43 and Asp 128, and the valeryl carboxylate is recognized by Ser 88 and Asn 49 via hydrogen bonds. In addition, several streptavidin residues (Val 47, Trp 79, Thr 90, Trp 92, Trp 108 and Leu 110) form a hydrophobic pocket and make Van der Waals contact. Peptides containing the HPQ motif bind to streptavidin in the biotin binding site (Figure 1B-1C). The histidine residue in the peptide forms a hydrogen bond with Ser 88 as did the carboxylate of biotin. Ser 45, which was involved in biotin binding, interacts with the proline carboxyl. Thr 90 of streptavidin forms ahydrogen bond with the peptide glutamine although this residue made hydrophobic contact with biotin. Differences between these binding modesmay result in the lower observed affinity.

Interactions outside the peptide HPQ consensus and differences in the binding pockets of the biotin receptors perhaps lead to target specificity, and, thus, lack of universal mimicry. For example, the linear peptide, FSHPQNT [12], forms additional hydrogen bonds between the asparagine residue of the peptide and Ser 27 and Asn 23, both of which are involved in interaction with the biotin urea group (Figure 1B). In addition, the binding pockets of the various biotin receptors have structural differences. For example, the biotin binding pocket of avidin includes an additional aromatic residue (Phe 72) which does not exist in streptavidin [19]. Furthermore, avidin does not contain a residue corresponding to streptavidin Ser 45 which is an important residue for binding biotin and the peptides. These differences may be the reason for the preference of the biotin mimicking peptides for streptavidin.

Although perhaps not universal biotin mimetics, streptavidin and avidin binding peptides have a myriad of applications. When fused to proteins they can be employed as affinity tags for the purification of recombinant proteins [20,21,22]. In addition to purification, these peptides have the potential to be utilized as probes for monitoring recombinant protein production and for sensitive bioassays [23,24]. More generally, the discovery of streptavidin binding peptides selected from combinatorial libraries demonstrated that it is possible to select high affinity ligands towards proteins which naturally bind to non-peptidic small-molecules.

## 3. Sugar Mimics

Molecular recognition events involving carbohydrates play a role in many biological processes from pathogen infection and tumor invasion to fertilization and cellular adhesion [27,28,29]. In addition, as carbohydrates are often displayed on the outer surfaces of pathogens and tumor cells, they could play a key role as novel immunological targets for diagnosis, antibody production and vaccine development. 

However, many of the carbohydrate potential antigens are only weakly immunogenic as they suffer from the inability to generate T-cell responses and lack specificity as they usually bind to multiple receptors. Therefore being able to manipulate carbohydrate interactions could have a clear impact in medicine and the pharmaceutical sciences. However advancement in this field is restricted by the challenges associated with carbohydrate synthesis. Much research has been undertaken to generate carbohydrates with stereo-control and high yields [30,31,32]. However, these procedures are still far from being routine for general researchers as the stereo- and regio-chemistry are difficult to control, and the products are polar and difficult to visualize thus resulting in purification challenges. Moreover, they often require tedious and inefficient cycles of functional group protection and deprotection reactions. Solid phase methods [33,34] have been established, but they still lack the complete generality and robustness to achieve wide-spread adoption.

A number of small-molecule based approaches to establish sugar mimics have focused on hanging hydroxyl functionality off a small, central scaffold [35,36]. The structural similarities of these scaffolds to sugars aid in their rational advancement to new systems and therefore have a clear advantage. However, their utility can be limited when applying them to combinatorial chemistry. Like most small-molecule libraries based around a single scaffold, their chemical and 3-D spatial projection diversity is limited, and requirements for deconvolution of resulting hits can further limit coverage of chemical space. Moreover, reliance on rational structural mimicry can impede the discovery of non-structurally related functional mimics. On the other hand, the high-yield, solid phage synthesis of peptides can be easily automated and the modification of peptides to increase immunogenicity is routine. However, how a peptide could mimic a sugar is not immediately obvious, therefore requiring the use of combinatorial libraries. Genetically encoded peptide libraries, like those afforded by phage display and which have clear advantages in library size and straight-forward tagging, can be complimentary to these small-molecule approaches to sugar mimicry. Therefore, multiple studies have focused on identifying sugar mimetic peptides through phage display screening against sugar-binding proteins and sugar-specific antibodies.

The first attempts to establish sugar-mimetic peptides using phage display were by biopanning against the jack bean lectin, concanavalin A (ConA). Scott *et al.* [37] used a linear hexapeptide library displayed on filamentous phage, f1, and identified the consensus sequence YPY among the isolated phage that bound ConA. It was speculated that phenolic hydroxyls of the tyrosines mimic those on the sugar and the hydrophobic nature of the sequence could be providing contacts similar to the ring carbons. The strongest binding peptide, MYWYPY, inhibited the binding of methyl *α*-D-mannopyranoside (*α* -ManOMe) to ConA indicating that it is interacting with the protein at or near the sugar-binding site. However, relative affinity measurements using ELISA with the individual clones suggested that binding to other *α*-ManOMe-binding lectins, *Lens culinaris* agglutinin and *Pisum sativum* agglutinin, is reduced by one to two orders of magnitude compared to ConA suggesting that the sugar mimicking role of these peptides is limited. Interestingly, under these ELISA conditions, a control phage exhibited a similar trend and was competitive with *α*-ManOMe. Concurrently, Oldenburg *et al.* [38] screened an octapeptide library fused to pIII of the fd bacteriophage. They eluted bound clones with either *α*-ManOMe or citrate buffer. The resulting clones converged on the YXY motif, and many of the clones eluted with *α*-ManOMe possessed YPY.One of these clones had a tandem repeat of YPY. This sequence was synthesized as a peptide fused to four residues from pIII to enhance solubility. The resultingdodecapeptide, DVFYPYPYASGS had a K_D_ of 46 µM which is comparable to *α*-ManOMe (89 µM). Interestingly the peptide exhibited an order of magnitude higher IC_50_ than that for *α*-ManOMe when inhibiting the precipitation of dextran with ConA which may be a consequence of the altered binding kinetics of the peptide [8]. In later studies this peptide was shown to inhibit ConA-induced T-cell proliferation [39].Furthermore it was shown that the peptide could react with anti-*α*-ManOMe polyclonal antibodies and *α*-ManOMe could bind anti-DVFYPYPYASGS antibodies thus strongly supporting the success of the peptide in mimicking the sugar [40]. 

**Figure 2:**
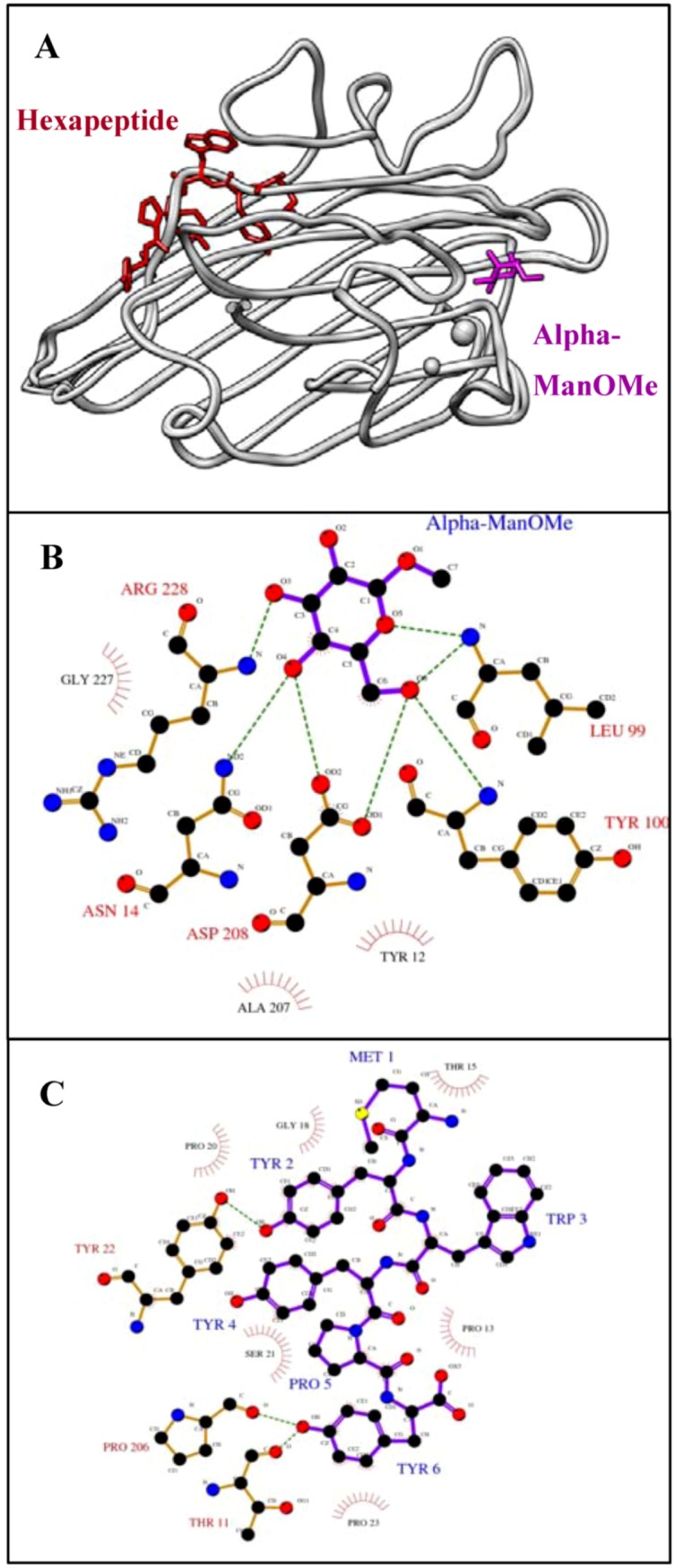
The sugar and its mimicking peptide bind different sites of ConA. **A)** Superposition (using the SoperPose server [59]) of the ConA/α-ManOMe [60] and ConA/hexapeptide (MYWYPY) complexes [61]. The molecular graphics image was produced using the UCSF Chimera package from the Resource for Biocomputing, Visualization, and Informatics at the University of California, San Francisco (supported by NIH P41 RR-01081) [62]. Ligplot analysis [25] to show the interactions between ConA and **B)** α-ManOMe, and **C)** the hexapeptide, MYWYPY. Hydrogen bonds are shown as green dashed lines and hydrophobic interactions are shown as red arcs. Key protein residues are labeled in black (hydrophobic) or red (hydrogen bonds) and peptide residues in blue. Because this is a two-dimensional representation, actual distances, bond lengths and spatial organization are distorted.

Structural studies [39,41], however, provide a conflicting story. Crystal structures demonstrate that the peptide binds ConA at a nearby, but distinct, binding site from that of the sugar (Figure 2A). The residues Asn 14, Leu 99, Tyr 100, Asp 208 and Arg 228 all form hydrogen bonds with mannose (Figure 2B). Several other residues, namely Tyr 12, Ala 207 and Gly 227, form hydrophobic interactions near C4 and C6 of the sugar. On the other hand, the MYWYPY peptide is bound to ConA in a pocket that is formed by different residues (Figure 2C). The hydroxyl group of tyrosine, at position 2 of the hexapeptide, makes a hydrogen bond with Tyr 22. Similarly, the C-terminal phenolic tyrosine hydroxyl, makes a hydrogen bonds with Thr 11 and Pro 206. In addition, the hexapeptide is surrounded by a hydrophobic surface made up of Thr 15, Pro 13, Gly 18, Pro 20, Pro 23 and Ser 21. These residues are not involved in the sugar interaction. Although, α-ManOMe and its peptide mimic bind ConA at nearby binding sites, not even one common interacting residue is shared. It has been postulated that the competitive nature of the peptide’s activity and its seemingly sugar-mimicking behavior are a consequence of the plasticity of antigen binding sites in the antibody and either overlap or some sort of allosteric communication between the two Con A sites [42]. 

Phage display has also been used to discover potential mimics of the complex carbohydrate, sialy Lewis X which, through its interaction with the mammalian lectin E-selectin, is involved in the early recruitment of neutrophils to injured or inflamed tissue. Martens *et al.* [43] used a phagemid approach to screen a number of libraries of peptides fused monovalently to pIII against E-selectin. Panning with both linear X_8_ and cyclic, variable size, CX_2-6_C peptide libraries resulted in no binding clones. Screening with a linear X_12_ library generated as a single convergence, HITWDQLWNVMN. Secondary screens of libraries based on this sequence using phage or peptides bound to plasmids through lac repressor fusions [44] were conducted and ultimately resulted in DITWDQLWDLMK. This peptide was able to inhibit the binding of leukocytes to immobilized E-selectin and could block an acute inflammation response upon intravenous treatment in mice. In addition, this peptide demonstrated remarkable affinity to E-selectin in a competition binding assay with a radio-labeled version of itself (Kd = 4 nm). However, unlike the natural interaction, this binding was not calcium dependent, and the peptide did not compete with the natural ligand for binding to the lectin, suggesting that the peptide may be operating through a mechanism distinct from that of the sugar. 

To discover mimics of the tumor-associated antigen Lewis Y oligosaccharide, Hoess *et al.* [45] screened a linear octapeptide phage display library against a monoclonal antibody specific for the antigen. Twenty selected phage isolates from the second and third round of biopanning all yielded the same peptide sequence, APWLYGPA. The synthesized peptide inhibited the binding of the antibody to its epitope expressed on A431 cells. In addition, the antibodies produced by immunizing with the peptide react with Lewis Y demonstrating that phage display derived peptides can function as sugar immunogens [46]. Further mechanistic studies demonstrated that the PWLY four residue block of the peptide is essential for its activity, and because of its similarity with YPY, two aromatic residues separated by one that is hydrophobic has been proposed as a common motif for sugar-mimicking peptides.

A number of additional studies have established immunogenic properties of phage display peptides which mimic the capsular polysaccharides of pathogenic microorganisms. Targeting for mimicry of the glucuronoxylomannan on the polysaccharide capsule of *Cryptococcus neuformans*, a fungus that causes meningoencephalitis in AIDS patients, was attempted by Valadon *et al.* [47], who screened deca- and hexa-peptide phage libraries against a mannan-selective monoclonal antibody. The affinity matured library converged on a number of motifs including TPXW[M/L][M/L]. One synthesized peptide, LQYTPSWMLV possessed high affinity for the antibody (*K*d = 295 nM) measured in an SPR-based binding assay. This binding was competitive with sugar ligand, suggesting that the peptide binding site overlaps with the polysaccharide binding site which was later supported by a crystal structure of peptide-antibody complex [48]. However, the binding mechanism of the peptide and the carbohydrate are somewhat divergent suggesting that the peptide would not be successful in generating an immune response against the sugar. 

Harris *et al.* [49] screened eleven monovalent VIII phage display libraries (X_6_, X_15_, X_30_, X_8_CX_8_, X_15_CX, XCX_15_, XCX_4_CX, XCX_6_CX, XCX_6_CX (with no M or W), XCX_8_CX, XCCX_3_CX_5_C) [50] against a number of antibodies selective for the cell-wall polysaccharide of group A *Streptococcus*. While many of the resulting convergent peptides were competitive with the carbohydrate for binding the antibodies, they showed little activity against the antibodies to which they were not selected against, suggesting that these ligands bind the antibodies with different mechanisms and therefore the peptides are not universally mimetic of the carbohydrate.

Similarly, Pincus *et al.* [51] sought to establish mimics of the group B streptococcal type III capsular polysaccharide (GBS) by screening a pIII fused nonameric peptide library against a selective monoclonal antibody. Strong convergence onto two sequences, WENWMMGNA and FDTGAFDPDWPAC, was observed. The second was demonstrated to bind the antibody to which it was selected along with other anti-GBS antibodies, and this binding was competitive with GBS. Immunization of mice with this peptide generated antibodies that were specific for GBS.

By screening a library made up of 15-mer peptides fused to pIII of the phage fd against monoclonal antibodies selective for the meningococcal group A polysaccharide (MAPS), Grothaus *et al.* [52] discovered a peptide, GEASGLCCRWSSLKGC, which was reactive with sera competitively with the MAPS, and generate an immune response that was active against MAPS. 

To establish mimics of the oligomannoside cell surface neutral polysaccharides (NPS) presented by *Mycobacterium tuberculosis*, Gevorkian *et al.* [53] screened a library of docomeric peptides fused to pIII of M13 against monoclonal anti-NPS antibodies. Panning resulted in clones that were active against antiserum and a number of consensus sequences including PLXGT[L/V]P.The peptide QEPLMGTVPIRA was able to generate an anti-NPS immune response upon immunization in rabbits. The anti-peptide antibodies were shown to be reactive against the high mannose tuberculosis lipoarabino-mannan (LAM). An interesting control experiment with these antibodies demonstrated that they were active against yeast mannan which is also mannose-rich, suggesting that the peptides are mimicking mannose.

The Gram negative bacterium *Shigella flexneri* is a human pathogen that is responsible for causing dysentery. To discover a peptide mimic of the O-antigen (O-Ag) of the lipopolysaccharide (LPS) of *S. flexnaeri*, Phalipon *et al.* [54] screened linear and disulfide-constrained cyclic nonapeptide libraries fused to pVIII of M13.Although no clear consensus was evident, a preference for aromatic residue was observed with 82% of the nineteen resulting clones having at least two of these residues, 55% having at least three and one possessing five. Two phage clones displaying the cyclic peptides, CYKPLGALTHC and CKVPPWARTAC, were used to immunize mice and the resulting antibodies have activity against LPS. These antibodies could be used to specifically label the pathogen as evidenced by fluorescent microscopic imaging.

Sugar mimicking phage displayed peptides are also promising candidates as therapeutic agents. The ganglioside GD1 *α* is involved in the metastatic adhesion of lymphoma cells to the hepatic sinusoidal endothelium (HSE). Ishikawa *et al.* [55] screened a 15-mer peptide library fused to pIII against a monoclonal antibody selective for the ganglioside. Although no consensus was observed, the resulting clones seemed to have a preference for aromatic and basic residues, and these all selectively bound to the antibody. To some extent all were competitive with GD1 *α*. The phage clone, displaying the peptide WHWRHRIPLQLAAGR, could be used to selectively image HSE cells and inhibited, as could the synthetic peptide, the binding of a lymphoma cell line to HSE. This peptide was able to reduce metastasis *in vivo* in lung, liver, and spleen upon mouse injection with lymphoma cells.

The Gal-α(1,3)Gal (α-Gal) epitope is displayed on porcine cells and its immunogenicity has reduced the utility of using pig organs for xenoplantation in humans. Lang *et al.* [56] panned an X_7_ linear and a CX_7_C cyclic peptide library against an α-Gal-specific antibody.The resulting clones converged on PTXSTL and were competitive with carbohydrate in their binding to the antibody thereby showing a therapeutic potential in xenoplantation. The cyclic peptide affinity could be decreased by disulfide reduction. The peptides and phage clones were subjected to porcine red blood cell agglutination assays with the phage demonstrating enhanced activity presumably due to the multivalent effect [7,8].

In summary, many studies have been undertaken to establish peptide mimics of sugars using phage display. Although the targets for mimicry, the nature of the receptors, and the resulting ligands are all quite diverse, some general principles are evident. Many of the convergent sequences possess aromatic residues, residues that display a hydroxyl (Ser, Thr, Tyr), and/or proline residues. The hydroxyl residues could be mimicking the hydroxyls of the sugars. The prolines could be inducing a sharp change of direction by allowing a cis peptide bond in the backbone so as to project functionality into a tighter three-dimensional zone much like a functional group dense sugar. And the aromatic residues could be π-stacking to the aromatic residues found in many sugar binding pockets that have been shown to interact with the sugar axial methine hydrogens or make cation-π-like interactions with the electron poor anomeric carbon or adjacent resonance-stabilized oxygen [57] and therefore may not afford much sugar specificity. In addition, these ligands interact with the receptors for which they have been selected, but many of them have not been assayed against other receptors that also bind the same sugar. In other words, in many cases, their universal mimicry has not been firmly established. As these screens are often designed to select for only the strongest binder, there often is no selective pressure to discover a ligand that binds all the receptors. 

To overcome this limitation, Yu *et al.* [58] recently developed a unique strategy for the sequential screening of a phage displayed peptide library against a set of multiple targets that bind to the same sugar ligand. The idea is that by linking different targets in the same round of a screen, the library can be matured to select for ligands that bind all the targets, and not simply for ligands that have a high affinity against one. Presumably this could be linked to negative screens to weed out binders that mimic structurally related small-molecules with very subtle differences, like glucose and galactose for example. The selected peptides demonstrated affinity to the multiple targets and were predicted to bind to the sugar binding site over the peptide binding site. However, their structural mimicry at atomic resolution needs to be further explored. It is possible that application of this philosophy to design screens for universal small-molecule mimics could be applied to other systems.

## 4. Conclusions

The past twenty years have demonstrated that phage display technology is a powerful tool for identifying peptide mimics of small-molecules. One of the most successful examples is the discovery of biotin mimetics. Although no universal mimic has yet been established, they have some advantages over natural ligand when used as affinity tags in biological applications as their affinity can be tuned and they can be expressed as fusions to recombinant proteins. 

Peptides that mimic carbohydrates have the potential to aid in developing new vaccines and therapeutics as they can generate T-cell dependent immune responses and can be easily synthesized. Mimetics established through phage display have been shown to be highly immunogenic and can induce anti-saccharide antibody production *in vivo* which is a key step in vaccine development. A further strength is that, phage display peptides libraries, by screening against antibodies against pathogens, can be employed to generate immunogenetic peptides that structurally mimic undefined carbohydrate antigens. Like the biotin mimetics, however, most of the sugar mimetic peptides discovered through phage display screens, have specificity only to the target used in biopanning. However, it is possible that this lack of universal mimicry could be overcome by the use of screens designed to pan multiple targets sequentially. Learning how to establish true universal mimicry of a specific small-molecule and expanding these techniques to other small-molecules are the next steps in this active field.
